# Lower levels of uric acid and striatal dopamine in non-tremor dominant Parkinson's disease subtype

**DOI:** 10.1371/journal.pone.0174644

**Published:** 2017-03-30

**Authors:** Ismael Huertas, Silvia Jesús, José Antonio Lojo, Francisco Javier García-Gómez, María Teresa Cáceres-Redondo, Juan Manuel Oropesa-Ruiz, Fátima Carrillo, Laura Vargas-Gonzalez, Juan Francisco Martín Rodríguez, Pilar Gómez-Garre, David García-Solís, Pablo Mir

**Affiliations:** 1 Unidad de Trastornos del Movimiento, Servicio de Neurología y Neurofisiología Clínica, Instituto de Biomedicina de Sevilla (IBiS), Hospital Universitario Virgen del Rocío/CSIC/Universidad de Sevilla, Seville, Spain; 2 Servicio de Medicina Nuclear. UDIM. Hospital Universitario Virgen del Rocío, Seville, Spain; 3 Centro de Investigación Biomédica en Red sobre Enfermedades Neurodegenerativas (CIBERNED), Madrid, Spain; Oslo Universitetssykehus, NORWAY

## Abstract

Parkinson’s disease (PD) patients who present with tremor and maintain a predominance of tremor have a better prognosis. Similarly, PD patients with high levels of uric acid (UA), a natural neuroprotectant, have also a better disease course. Our aim was to investigate whether PD motor subtypes differ in their levels of UA, and if these differences correlate with the degree of dopamine transporter (DAT) availability. We included 75 PD patients from whom we collected information about their motor symptoms, DAT imaging and UA concentration levels. Based on the predominance of their motor symptoms, patients were classified into postural instability and gait disorder (PIGD, n = 36), intermediate (I, n = 22), and tremor-dominant (TD, n = 17) subtypes. The levels of UA and striatal DAT were compared across subtypes and the correlation between these two measures was also explored. We found that PIGD patients had lower levels of UA (3.7 vs 4.5 vs 5.3 mg/dL; P<0.001) and striatal DAT than patients with an intermediate or TD phenotype. Furthermore, UA levels significantly correlated with the levels of striatal DAT. We also observed that some PIGD (25%) and I (45%) patients had a predominance of tremor at disease onset. We speculate that UA might be involved in the maintenance of the less damaging TD phenotype and thus also in the conversion from TD to PIGD. Low levels of this natural antioxidant could lead to a major neuronal damage and therefore influence the conversion to a more severe motor phenotype.

## Introduction

Parkinson’s disease (PD) patients can be categorized, based on the predominant motor symptoms, into tremor-dominant (TD) and non-tremor dominant subtypes[1]. The former is characterized by a predominance of tremor and the latter by a predominance of rigidity, akinesia, and/or postural instability and gait disorder (PIGD) symptoms. This distinction differs between symptom predominance at presentation of the disease and symptom predominance at later stages, since some patients presenting with tremor at onset become predominantly rigid-akinetic or PIGD in more advanced stages[2]. Previous studies have demonstrated that PD patients who continue to have tremor dominance after several years progress more slowly than those with predominance of non-tremor symptoms[3, 4], including a lower likelihood to develop cognitive impairment and dementia[5, 6]. At autopsy, TD patients have less pathological burden[7, 8], and accordingly, neuroimaging studies have found a higher degree of dopaminergic denervation[9–13] and grey matter atrophy in the non-tremor subtype[14]. It is not yet well-known whether the pathophysiological mechanisms underlying these PD subtypes are different[15, 16].

A major challenge is to unravel why PD patients presenting and maintaining a TD phenotype have a better prognosis (i.e. slower deterioration), or conversely, why PD patients with a non-tremor onset, or PD patients with a tremor onset but converting to rigid-akinetic and/or PIGD, progress worse (i.e. faster deterioration). It is likely that multiple factors play a role in PD phenotype and progression, including genetic susceptibility loci and environmental factors, but the identification of potential biomarkers is essential to improve treatment. A possible explanation for these differences could be that, although the biological mechanisms triggering sporadic PD for both subtypes were the same, these patients might differ in their levels of endogenous neuroprotective agents. In this regard, uric acid (UA) is an important natural antioxidant in the human body and it has been demonstrated that it reduces damage to neurons caused by oxidative stress. Indeed, low levels of serum UA have been associated with an increased risk to develop neurodegenerative diseases, including PD[17, 18]. Furthermore, longitudinal studies have determined that PD patients with lower levels of serum UA deteriorate more quickly[19, 20].

In this study we sought to investigate whether the tremor and non-tremor PD subtypes differ in their levels of serum UA and degree of dopaminergic degeneration, and if these two measurements correlate. To this end, we collected motor information, UA levels and dopamine transporter (DAT) imaging by [^123^I]FP-CIT SPECT from 214 PD patients. Using retrospective data from the disease motor onset, we investigated in late stage patients whether patients who consistently maintained throughout the time a TD phenotype had higher values of UA as compared with those who developed PIGD symptoms. We hypothesized that the major neuronal damage seen in non-tremor PD patients could be related to a lower degree of neuroprotection, and UA might be one of the factors implicated in these mechanisms.

## Materials and methods

### Subjects

We collected information from75 PD patients (60% males, age of onset 46 ± 10 years [19–64], disease duration after symptom onset: 12 ± 6 years [5–23]) whom visited the Movement Disorders Unit from2004 to 2014 at Hospital Virgen del Rocío (Seville, Spain). All patients had confirmed PD diagnosis based on the UK Parkinson's Disease Society Brain Bank clinical diagnostic criteria. For all these patients, we collected information about motor status and symptoms, [^123^I]FP-CIT SPECT, and serum UA concentration levels. The study was approved by the Hospital Virgen del Rocío ethics committee and all patients signed informed consent to participating in the study.

### Uric acid measurement

Blood samples of the patients were processed after extraction at the Central Laboratory of our Hospital. Serum urate concentration was determined by means of enzymatic assay following the standards in routine clinical practice. We did not include patients with conditions that could affect the serum UA concentration such as lymphoproliferative diseases, cancer, chemotherapy, haemolytic and pernicious anaemia, alcoholism, hypoxanthine phosphorylated guanidine deficits, gout, diabetic and alcoholic ketoacidosis, treatments that could affect the serum UA concentration [acetylsalicylicacid, diuretics, nicotinic acid, ethambutol, allopurinol, cyclosporine and pyrazinamide], lead poisoning, renal disease and hypo- or hyperthyroidism.

### Classification into motor subtypes

Patients were classified based on established methods into TD, PIGD, or intermediate (I) using items from the Unified Parkinson’s Disease Rating Scale (UPDRS), off-medication[3].We considered that the motor phenotype at this UPDRS evaluation after a mean follow-up of 12 years was stable and definite. Patients’ tremor score was determined by adding items 16 and 20–21, and dividing by 8, and balance and gait score by adding items 13–15 and 29–30, and dividing by 5. Patients were defined as TD if the ratio of the tremor score divided by the balance and gait score was ≥ 1.50, PIGD if the ratio was ≤ 1, and as I if the ratio was between 1–1.50. Disease severity by Hoehn and Yahr (H&Y) stage and the main symptom at onset (Tremor Onset (TO): any presence of tremor (from slight to severe) vs. Non-tremor Onset) were also recorded.

### SPECT imaging

The acquisition procedure and SPECT reconstruction were carried out following standard protocols and can be found in a previous report[21].SPECT images were processed with SPM8 using a homemade [^123^I]FP-CIT template (http://www.nitrc.org/projects/spmtemplates), and following the standard processing pipeline in SPM. We extracted the average [^123^I]FP-CIT binding at posterior and anterior putamen, and posterior caudate (head of caudate) using a custom-made parcellation of the striatum (https://www.nitrc.org/projects/striatalvoimap) following established methodology[22].For each patient and striatal subregion, we calculated the [^123^I]FP-CIT specific binding ratio (SBR) with respect to a non-specific volume in the occipital cortex by the formula: (striatal regional binding—occipital binding)/occipital binding.

Given the high influence of age and scanner in the value of SBR [23], we have worked with the age-normalized SBR instead (nSBR) (although raw SBR are also provided in supporting information). This measure is defined as the ratio between the patient SBR and the average age-specific healthy SBR, the latter calculated from linearly regressing age and SBR of normal controls. Thus, this measure represents the age and scanner-specific reduction of SBR due to the disease and makes these imaging variables more comparable with other studies using other machines [24]. For this, we used in-house images of 184 scans without evidence of dopaminergic deficit from the same scanner (age range 18–90 years; 56% males). In line with previous findings, we observed a 5.1% SBR decline per decade in these normal controls [23]. Lastly, since PD is asymmetrical by nature and laterality can affect the statistics at the group level, the comparisons were made for contralateral and ipsilateral to the most affected side instead of right and left.

### Statistical analysis

We compared the three motor subtypes (TD, I and PIGD) using Chi-square test for nominal variables, ANOVA for quantitative variables, and the Kruskal Wallis test for the H&Y stage. Post-hoc analyses were performed to compare significant associations in a pairwise manner. Additionally, since UA levels are higher in men than in women by nature, we examined UA levels separated by sex as well. The correlation between UA and SPECT variables was evaluated with Pearson’s coefficient and the value of R^2^ (explained variance) is reported. Linear regression analyses were also performed to verify that the associations of UA and striatal nSBR with the motor subtype were not confounded by other variables such as age, sex and disease severity (UPDRSIII and H&Y). The significance threshold was set to P<0.05. Statistical analyses were performed with IBM SPSS Statistics 22.0.

## Results

**Fig 1:**
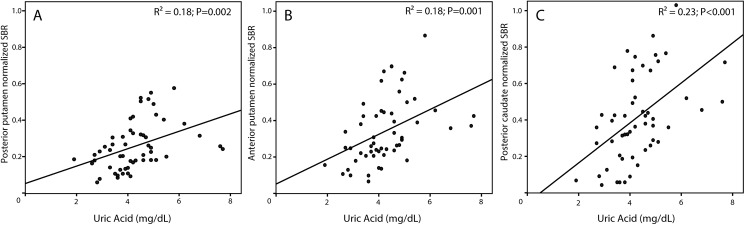
**Correlation of uric acid and contralateral normalized specific binding ratio (nSBR) for posterior putamen (A), anterior putamen (B), and posterior caudate (C)**.

Descriptive values and statistics are shown in Table 1. Of the 75 PD patients, 36 were classified as PIGD, 22 as I and 17 as TD. ANOVA indicated that UA levels were different across subtypes (P<0.001). Post-hoc analyses revealed that UA was significantly lower for PIGD than for TD (3.7 vs 5.3; P<0.001) and I (3.7 vs 4.5; P = 0.05) subtypes. When segregated by sex, UA levels were slightly higher in males as expected, but importantly, the differences across motor subtypes remained consistent (P = 0.041). Post-hoc comparisons were still significant between PIGD and TD subtypes although the differences between PIGD and I subtypes vanished probably due to a loss in statistical power. ANOVA analyses also showed differences in striatal nSBR across motor subtypes (P<0.01, see Table), and in particular that PIGD had lower nSBR values than TD and I phenotypes (P<0.05). This was consistent for both ipsilateral and contralateral hemispheres and for both normalized SBR and raw SBR (raw SBR values are available in S1 Table in supporting information). Furthermore, UA levels were significantly correlated with striatal nSBR in posterior putamen (R^2^ = 0.17; P = 0.002), anterior putamen (R^2^ = 0.18; P = 0.001) and posterior caudate (R^2^ = 0.23; P<0.001) (Fig 1).

**Table 1:** Demographic values, motor information, uric acid levels and the regional age-normalized [^123^I]FP-CIT SBR.

	PIGD (n = 36) (n = 36)	I (n = 22)	TD (n = 17)	p-value
Sex (m/f)	17 / 19	15 / 7	13 / 4	N.S.
Age of onset (y)	46 ± 9	41 ± 9	45 ± 12	N.S.
Disease duration (y)	13 ± 6	13 ± 6	11 ± 7	N.S.
TO (%)	25%	45%	100%***	**<0.001**
UPDRS-III	49 ± 12	49 ± 12	46 ± 11	N.S.
UPDRS Total	90 ± 22	86 ± 22	74 ± 14*	**0.036**
Hoehn &Yahr	4 [3, 5]	3 [2.5, 4]***	3 [2.5, 3]***	**<0.001**
Uric Acid (mg/dL)	3.7 ± 0.9	4.5 ± 1.1*	5.3 ± 1.3***	**<0.001**
Male	4.0 ± 1.0	4.7 ± 1.0	5.3 ± 1.3*	**0.041**
Female	3.4 ± 0.8	4.0 ± 1.0	5.2 ± 1.8*	**0.041**
Posterior putamen nSBR				
contralateral	0.18 ± 0.13	0.29 ± 0.14*	0.30 ± 0.16*	**0.004**
ipsilateral	0.24 ± 0.13	0.34 ± 0.15*	0.38 ± 0.18**	**0.004**
Anterior putamen nSBR				
contralateral	0.24 ± 0.16	0.38 ± 0.18*	0.40 ± 0.20*	**0.002**
ipsilateral	0.31 ± 0.16	0.45 ± 0.19*	0.47 ± 0.20**	**0.002**
Posterior caudate nSBR				
contralateral	0.28 ± 0.25	0.49 ± 0.28*	0.46 ± 0.29*	**0.008**
ipsilateral	0.40 ± 0.26	0.63 ± 0.29*	0.61 ± 0.29**	**0.002**

* p<0.05

** p<0.01

*** p<0.001 for post-hoc analyses with respect to PIGD.

PIGD: postural instability and gait disorder subtype; TD: tremor-dominant subtype, I: intermediate subtype; TO: Tremor Onset; N.S.: not significant.

Regarding motor scales, PIGD patients were more severely affected than TD and I patients (H&Y = 4 > 3, P<0.001), and had UPDRS total score was higher than TD patients (90 vs 74, P<0.05).Multivariate regression analyses confirmed that the positive univariate associations between motor subtypes and UA (β = 0.43, P = 0.006) and (putamen) nSBR (β = 0.38, P = 0.01) remained significant after correcting for the potential confounds and the differences in severity. Finally, when retrospectively examined the motor symptom onset of these patients, we observed that all TD patients (100%) had a tremor onset, but there was also anon-negligible percentage of patients with tremor onset who converted to I (45%) and to PIGD (25%) over the disease course. In this article, we speculate that this conversion may be partially driven by UA.

## Discussion

In this study we found that PD patients with a PIGD motor phenotype have lower levels of serum UA and striatal DAT availability than PD patients with a predominance of tremor, and that the levels of UA are correlated with the degree of striatal dopamine depletion. These associations were significant in both univariate and multivariate analyses where potential confounds were also accounted for. Furthermore, using retrospective data of late stage patients, we also found that patients who maintained a TD phenotype throughout the disease course, or to a lesser extent progressed to an intermediate phenotype, had higher levels of UA and striatal DAT than PD patients who converted to the PIGD phenotype.

Our results are in line with a similar study that found significant lower UA levels and higher disease severity in PD patients with a non-tremor subtype[25]. These authors also found consistent differences across motor subtypes for both genders, which is important given the inherent difference in UA values between men and women. However it is worth commenting that the levels of UA concentration in the study of Lolekha and colleagues were in overall higher than in our study. For example, on average, they observed 5.9 mg/dL in their TD patients as compared with 5.3 mg/dL in our cohort, and these differences were even more accentuated when segregated by sex, where they report an average value of 6.4 mg/dL for TD males. We discard the UA assessment method as the main cause of this discrepancy since we also followed standard methodology to measure UA concentration, so we speculate that these differences are more likely due to intrinsic differences between the two populations (Thailand vs Spain) such as diet and ethnic origin among others. Also in line,our group in a previous study, and another cross-sectional study found lower levels of UA in advanced Hoehn and Yahr stages[26, 27].The longitudinal PRECEPT and DATATOP studies also found that PD patients with lower levels of UA at baseline suffered from faster rates of clinical progression, including greater declines in the UPDRS total score and striatal DAT availability[19, 20].

Imaging of the striatal DAT is also a marker of disease severity and PD subtypes show a different degree of dopaminergic depletion in both hemispheres. In particular, higher motor severity, postural instability and falling have been associated with lower striatal DAT binding[28].Previous studies also found higher [^123^I]FP-CIT uptake in TD in both putamen and caudate than in rigid-akinetic [12, 13]. A longitudinal study found higher declines in rigid-akinetic in striatal DAT at follow-up, although they found no differences at baseline[9]. Rossi et al. only found differences in putamen, probably because they included patients at a very early stage (<1 year of disease duration) and the caudate region was probably not sufficiently affected for differences between groups to be noted.[11] Similarly, our results are partially consistent with a recent study that found higher caudate [^123^I]FP-CIT uptake but no differences in putaminal binding[10]. However, since the putamen is the part of the striatum involved in the motor loop, it is surprising to find that patients with a different motor phenotype do not differ in their levels of dopamine depletion in this region. Some obvious differences in methodology could have led to this discordance.

The correlations between UA levels and DAT binding are consistent with a recent study performed in 52 newly diagnosed, drug-naïve PD patients, although its correlation coefficients (R^2^_putamen_ = 0.136; R^2^_caudate_ = 0.349) were not exactly the same[29]. These correlations are also in line with findings from the PRECEPT study, in which it was found that higher levels of urate were associated with a greater likelihood of a DAT scan without evidence of a dopaminergic deficit[30]. Of relevance, all of these results are in line with the hypothesis that UA acts as a protector against dopaminergic degeneration and, in keeping with this notion, a recent animal study found attenuated toxic effects on nigral dopaminergic cell counts and striatal dopamine content in UOx (the gene encoding urate oxidase) knockout mice[31].

**Fig 2:**
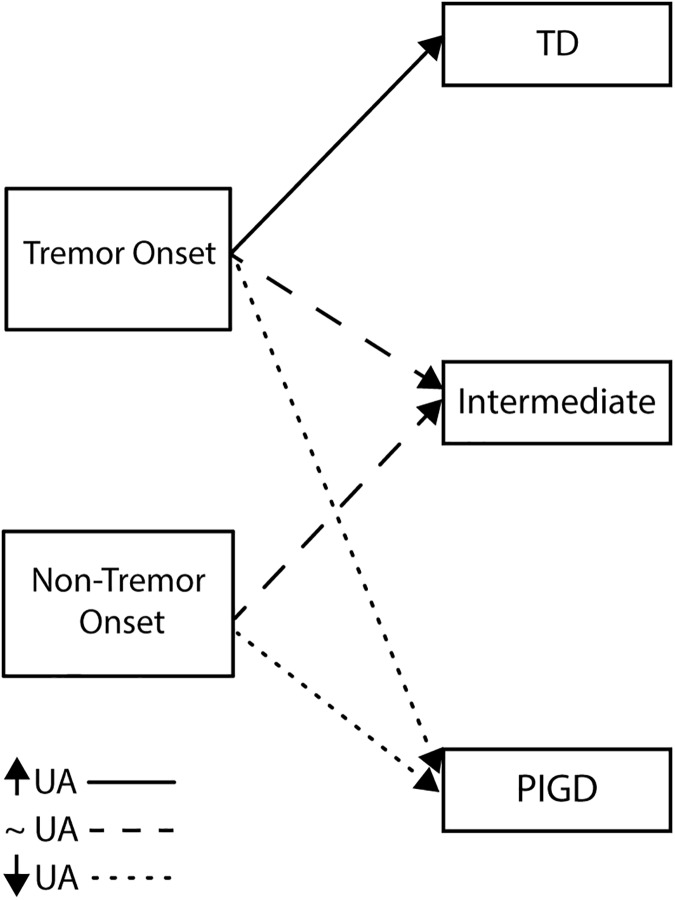
Diagram of conversion to motor phenotypes according to the levels of uric acid.

We observed that 100% of TD had a tremor onset, but there was also a non-negligible proportion of patients that converted from a tremor-dominant onset to an intermediate phenotype (45% of I) or even to PIGD(25% of PIGD). This suggests that, in accordance with the literature[2],PD patients with a tremor onset may evolve as the disease progresses to TD, I or, with a lower likelihood, to PIGD. Although a longitudinal study is necessary to corroborate this hypothesis, we speculate that those patients with a tremor onset that will maintain a TD phenotype may be those with higher levels of UA. Conversely, those with lower levels of UA could convert to an intermediate or PIGD subtype (Fig 2). Predicting motor disease progression is highly complex and may depend on multiple interacting factors (including genetic factors), but our results suggest that the motor phenotype may also be partially driven by UA levels. In this case, high levels of UA could help to maintain a less damaging TD form of PD.

Lastly, we acknowledge the limitations in our design and sample size to draw solid conclusions about our results. To corroborate our hypothesis, it would be very interesting to study in longitudinal cohorts such as PRECEPT the progression/conversion to motor phenotypes based on the levels of UA at baseline and at follow-up. Unfortunately, direct comparison is not possible since these researchers compared and reported an UPDRS total score obtained by adding the motor activity, mentation, and activity of daily living subscales and did not perform motor subtype classification.

In summary, in this study we provide further evidence about the differences in UA and striatal DAT between PD motor subtypes. We hypothesize and would like to test in the future whether PD patients who present with a tremor onset and maintain predominance of tremor, or, to a lesser extent, an intermediate phenotype, have higher levels of UA than those who develop PIGD symptoms. In this scenario, UA might be involved in maintaining the less damaging tremor-dominant form of PD. Low levels of this natural antioxidant may lead to a lesser degree of neuroprotection and could therefore influence motor phenotype and the clinical course.

## Supporting information

S1 Table Demographic values, motor information and the striatal subregional [^123^I]FP-CIT SBR. (DOCX)
