# Correction: Lower levels of uric acid and striatal dopamine in non-tremor dominant Parkinson's disease subtype

**DOI:** 10.1371/journal.pone.0176628

**Published:** 2017-04-26

**Authors:** The PLOS ONE Staff

There are errors in the funding section. The correct funding information is as follows: This work was supported by grants from the Instituto de Salud Carlos III-Fondo Europeo de Desarrollo Regional (ISCIII-FEDER) [PI14/01823, PI16/01575], the Consejería de Economía, Innovación, Ciencia y Empleo de la Junta de Andalucía [CVI-02526, CTS-7685], the Consejería de Salud y Bienestar Social de la Junta de Andalucía [PI-0437-2012, PI-0471/2013], the Sociedad Andaluza de Neurología, the Jacques and Gloria Gossweiler Foundation, the Fundación Alicia Koplowitz and the FundaciónMutuaMadrileña. Ismael Huertas was supported by the "PFIS" programme [FI14/00497], Silvia Jesús by the Rio Hortega programme, Juan Francisco Martin Rodríguez by the Sara Borrell programme, and Pilar Gómez-Garre by the Miguel Servet programme, all from the ISCIII-FEDER. The Publisher apologizes for the errors.
