# Supplementary material for: Lower levels of uric acid and striatal dopamine in non-tremor dominant Parkinson's disease subtype
Source: PLoS One. 2017 Mar 30;12(3):e0174644. doi: 10.1371/journal.pone.0174644 (PMC5373593; doi:10.1371/journal.pone.0174644)
Supplement: S1 Table — (DOCX) [file pone.0174644.s001.docx]

|  | PIGD (n=36) (n=36) | I (n=22) | TD (n=17) | p-value |
| --- | --- | --- | --- | --- |
| Sex (m/f) | 17 / 19 | 15 / 7 | 13 / 4 | N.S. |
| Age of onset (y) | 46 ± 9 | 41 ± 9 | 45 ± 12 | N.S. |
| Disease duration (y) | 13 ± 6 | 13 ± 6 | 11 ± 7 | N.S. |
| TO (%) | 25% | 45% | 100%^***^ | **<0.001** |
| UPDRS-III | 49 ± 12 | 49 ± 12 | 46 ± 11 | N.S. |
| UPDRS Total | 90 ± 22 | 86 ± 22 | 74 ± 14^*^ | **0.036** |
| Hoehn & Yahr | 4 [3, 5] | 3 [2.5, 4]^***^ | 3 [2.5, 3]^***^ | **<0.001** |
|  |  |  |  |  |
| Posterior putamen |  |  |  |  |
| contralateral SBR | 0.30 ± 0.20 | 0.44 ± 0.21^*^ | 0.44 ± 0.24^*^ | **0.02** |
| ipsilateral SBR | 0.39 ± 0.21 | 0.53 ± 0.23 | 0.55 ± 0.24^*^ | **0.02** |
| Anterior putamen |  |  |  |  |
| contralateral SBR | 0.50 ± 0.30 | 0.74 ± 0.37^*^ | 0.75 ± 0.37^*^ | **0.01** |
| ipsilateral SBR | 0.64 ± 0.35 | 0.87 ± 0.37^*^ | 0.88 ± 0.37^*^ | **0.02** |
| Posterior caudate |  |  |  |  |
| contralateral SBR | 0.28 ± 0.22 | 0.46 ± 0.28^*^ | 0.42 ± 0.25 | **0.02** |
| ipsilateral SBR | 0.40 ± 0.24 | 0.59 ± 0.30^*^ | 0.55 ± 0.24 | **0.02** |

**S1 Table**. Demographic values, motor information and the striatal subregional [^123^I]FP-CIT SBR.
